# Traité D’Hygiène Publique Et Privée

**Published:** 1845-09

**Authors:** 


### BIBLIOGRAPHICAL NOTICES.

*Traité d'Hygiène Publique et Privée,* par Michel Levy, Médecin Ordinaire de Première Classe et Professeur d'Hygiène et de Médecine Légale à l'Hôpital Militaire de Perfectionnement de Paris. 8vo. 2 vols., pp. 702, 796. Paris, 1844, 1845.

